# The relationship between amyloid pathology, cerebral small vessel disease, glymphatic dysfunction, and cognition: a study based on Alzheimer’s disease continuum participants

**DOI:** 10.1186/s13195-024-01407-w

**Published:** 2024-02-20

**Authors:** Hui Hong, Luwei Hong, Xiao Luo, Qingze Zeng, Kaicheng Li, Shuyue Wang, Yeerfan Jiaerken, Ruiting Zhang, Xinfeng Yu, Yao Zhang, Cui Lei, Zhirong Liu, Yanxing Chen, Peiyu Huang, Minming Zhang, for the Alzheimer’s Disease Neuroimaging Initiative (ADNI)

**Affiliations:** 1 Department of Radiology, School of Medicine, The Second Affiliated Hospital of Zhejiang University, Hangzhou, China; 2 Department of Neurology, School of Medicine, The Second Affiliated Hospital of Zhejiang University, Hangzhou, China

## Abstract

**Background:**

Glymphatic dysfunction is a crucial pathway for dementia. Alzheimer’s disease (AD) pathologies co-existing with cerebral small vessel disease (CSVD) is the most common pathogenesis for dementia. We hypothesize that AD pathologies and CSVD could be associated with glymphatic dysfunction, contributing to cognitive impairment.

**Method:**

Participants completed with amyloid PET, diffusion tensor imaging (DTI), and T2 fluid-attenuated inversion-recovery (FLAIR) sequences were included from the Alzheimer’s Disease Neuroimaging Initiative (ADNI). White matter hyperintensities (WMH), the most common CSVD marker, was evaluated from T2FLAIR images and represented the burden of CSVD. Amyloid PET was used to assess Aβ aggregation in the brain. We used diffusion tensor image analysis along the perivascular space (DTI-ALPS) index, the burden of enlarged perivascular spaces (PVS), and choroid plexus volume to reflect glymphatic function. The relationships between WMH burden/Aβ aggregation and these glymphatic markers as well as the correlations between glymphatic markers and cognitive function were investigated. Furthermore, we conducted mediation analyses to explore the potential mediating effects of glymphatic markers in the relationship between WMH burden/Aβ aggregation and cognition.

**Results:**

One hundred and thirty-three participants along the AD continuum were included, consisting of 40 CN − , 48 CN + , 26 MCI + , and 19 AD + participants. Our findings revealed that there were negative associations between whole-brain Aβ aggregation (*r* =  − 0.249, *p* = 0.022) and WMH burden (*r* =  − 0.458, *p* < 0.001) with DTI-ALPS. Additionally, Aβ aggregation (*r* = 0.223, *p* = 0.041) and WMH burden (*r* = 0.294, *p* = 0.006) were both positively associated with choroid plexus volume. However, we did not observe significant correlations with PVS enlargement severity. DTI-ALPS was positively associated with memory (*r* = 0.470, FDR-*p* < 0.001), executive function (*r* = 0.358, FDR-*p* = 0.001), visual-spatial (*r* = 0.223, FDR-*p* < 0.040), and language (*r* = 0.419, FDR-*p* < 0.001). Conversely, choroid plexus volume showed negative correlations with memory (*r* =  − 0.315, FDR-*p* = 0.007), executive function (*r* =  − 0.321, FDR-*p* = 0.007), visual-spatial (*r* =  − 0.233, FDR-*p* = 0.031), and language (*r* =  − 0.261, FDR-*p* = 0.021). There were no significant correlations between PVS enlargement severity and cognitive performance. In the mediation analysis, we found that DTI-ALPS acted as a mediator in the relationship between WMH burden/Aβ accumulation and memory and language performances.

**Conclusion:**

Our study provided evidence that both AD pathology (Aβ) and CSVD were associated with glymphatic dysfunction, which is further related to cognitive impairment. These results may provide a theoretical basis for new targets for treating AD.

**Supplementary Information:**

The online version contains supplementary material available at 10.1186/s13195-024-01407-w.

## Background

The glymphatic system connects perivascular spaces (PVS) over which cerebrospinal fluid (CSF) surrounding the brain exchanges with the interstitial fluid (ISF) within the parenchyma [1]. Its essential function is to take away wastes from the brain parenchyma. Detailly, CSF enters brain extracellular space via PVS, exchanges with ISF, and finally efflux via PVS again, taking away brain metabolic wastes. The failure of the glymphatic system is recently proposed as the final common pathway to dementia because it contributes to the aggregation of proteins, activates neuroinflammation and facilitates a positive feedback loop between wastes accumulation and further glymphatic dysfunction, and finally causes brain injury and dementia [2]. While the association between glymphatic dysfunction and dementia is widely acknowledged, few studies investigated factors related to glymphatic impairment.

Alzheimer’s disease (AD) pathologies, especially Aβ aggregation, were previously demonstrated to be related to glymphatic dysfunction in AD animal models [3–5]. While some studies suggested glymphatic dysfunction as the initial event precedes Aβ aggregation [3, 6], others found that glymphatic function compromised in the early stages of Aβ deposition in the APP/PS1 model [5, 7]. One recent study that used an in vivo imaging marker to evaluate the glymphatic function validated the relationship between Aβ accumulation and glymphatic dysfunction [8]. This study found that the impaired in vivo glymphatic marker preceded Aβ accumulation. Apart from AD pathologies, a damaged glymphatic function is also consistently reported in CSVD-related animal studies [9–11]. Hypertensive rats, the most common model of CSVD, showed suppressed glymphatic function [9], and the multiple microinfarct murine model also demonstrated global glymphatic pathway impairment [11]. In vivo studies using imaging markers for CSVD, mostly white matter hyperintensities (WMH) burden, found its spatial correlation with enlarged PVS and other non-invasive glymphatic markers [12, 13]. The effect of AD pathologies and CSVD on glymphatic function is separately proved in AD and CSVD models, respectively. Few studies have investigated the independent association of CSVD and AD pathologies with glymphatic function in vivo.

AD pathologies co-existing with CSVD are prevalent in aging and the most common pathogenesis for dementia. Of patients diagnosed with AD, > 80% were with CSVD [14].

Therefore, it is important to reveal the effects of Aβ and CSVD on glymphatic function from cognitively normal to dementia, if Aβ and CSVD independently contribute to glymphatic dysfunction or interactively damages glymphatic function.

To evaluate glymphatic function in vivo, we employed three non-invasive methods. Enlarged PVS is a commonly used approach to evaluate glymphatic function. It provides valuable information about the state of PVS, with severe enlargement indicating glymphatic dysfunction [15, 16]. The “Diffusion Tensor Imaging Analysis along the Perivascular Space” (DTI-ALPS) method, initially proposed by Taoka et al. [17], quantifies the diffusion of water within PVS along deep medullary veins and has robust correlations with glymphatic clearance, as determined by dynamic contrast-enhanced imaging [18]. Sleep, a crucial factor influencing glymphatic function, has been linked to DTI-ALPS [19, 20]. Furthermore, DTI-ALPS is closely associated with vascular risk factors [21, 22], which are linked to a decreased driving force for glymphatic function [9, 23, 24]. This method has also been applied to quantify in vivo glymphatic activity in various neurological disorders, including idiopathic normal pressure hydrocephalus (iNPH) [25], Parkinson’s disease [26], and multiple sclerosis [27] (MS), consistently revealing that reduced DTI-ALPS values are associated with disease severity. Choroid plexus volume, suggested as a driving force behind glymphatic function, has demonstrated associations with disease severity in conditions like frontotemporal dementia (FTD) [28] and AD [29] and has been used to predict conversion to Parkinson’s disease dementia [30].

Therefore, in this study, we aim to investigate (1) the association of CSVD and Aβ with glymphatic markers focused on AD continuum participants from cognitively normal to dementia and (2) whether glymphatic markers mediate the relationship between CSVD/Aβ and cognitive performance. CSVD was defined using WMH, a widely recognized and frequently utilized imaging marker for CSVD in the context of AD [31, 32]. We speculate that both WMH and Aβ are independently associated with glymphatic impairment and could damage cognitive performance via glymphatic dysfunction.

## Methods and materials

### Ethical approval

All procedures performed in studies involving human participants were under the ethical standards of the Institutional and National Research Committee and with the 1964 Helsinki declaration and its later amendments or comparable ethical standards. Written informed consent was from all participants and authorized representatives, and the study partners before any protocol-specific procedures were carried out in the ADNI study. More details can be found from http://www.adni-info.org.

### Participants

The data used in this article were from the Alzheimer’s Disease Neuroimaging Initiative (ADNI) database (http://www.loni.ucla.edu/ADNI). The database was launched by the National Institute on Aging (NIA), the Food and Drug Administration (FDA), and the National Institute of Biomedical Imaging and Bioengineering (NIBIB), aiming to explore whether serial magnetic resonance imaging (MRI), positron emission tomography (PET), biological markers, and other neuropsychological assessment can be used for early detecting and tracking AD. Ascertain of specific and sensitive markers for early AD will assist clinicians and researchers in understanding AD better and developing novel treatments.

The inclusion criteria were as follows: (1) completion of amyloid PET, diffusion tensor imaging (DTI) and T2 fluid-attenuated inversion-recovery imaging (FLAIR); (2) the interval between amyloid PET and DTI should be less than 12 months; (3) DTI should be acquired using the same parameters (seven images without diffusion weighting (*b* = 0 s/mm^2^) and 48 images with diffusion gradients were acquired (*b* = 1000 s/mm^2^)). Participants with a Geriatric Depression Scale (GDS) ≥ 5 and chin-up (DTI-ALPS is previously demonstrated to be sensitive to chin-up; it is defined as patients with AC-PC line over horizon line more than 20°) were excluded [33]. The information on participants with chin-ups was mentioned in Supplementary Material 1.

Vascular risk factors, such as hypertension, diabetes, hyperlipidemia, smoking, and heart diseases (including atrial fibrillation, atrial flutter, and coronary heart disease), were recorded based on participants’ medical history.

### Neuropsychological assessment

The neuropsychological assessments included general cognitive state: MMSE, memory function (ANDI-Mem), executive function (ADNI-EF), language (ADNI-Lan), and visuospatial function (ADNI-VS). Detailed information can be found in Supplementary Material 2. Clinical status was assessed by ADNI, categorizing subjects as cognitive normal (CN), mild cognitive impairment (MCI), and demented.

### MRI acquisition

Participants were from 17 different centers, and all of them underwent 3 T MRI scanning using SIMENS machine. T1-weighted images were obtained using a sagittal volumetric magnetization-prepared rapid gradient-echo (MPRAGE) sequence with the following representative imaging parameters: repetition time (TR) = 2300 ms, echo time (TE) = 3 ms, voxel size = 1.0 × 1.0 × 1.0 mm^3^, acquisition matrix = 256 × 240 mm^2^, flip angle = 9° or 11°. DTI images were obtained with the following imaging parameters: TR = 7200 ms, TE = 56 ms, voxel size = 2.0 × 2.0 × 2.0 mm^3^, acquisition matrix = 116 × 116 mm^2^. Seven images without diffusion weighting (*b* = 0 s/mm^2^) and 48 images with diffusion gradients were acquired (*b* = 1000 s/mm^2^). The T2 fluid-attenuated inversion-recovery (FLAIR) sequence was obtained using an echo-planar imaging sequence with the following parameters: TR = 4800 ms, TE = 441 ms, inversion time (TI) = 1650 ms, voxel size = 1.0 × 1.0 × 1.2 mm^3^, and acquisition matrix = 256 × 256 mm^2^. More information can be seen on the ADNI scanning website (http://adni.loni.usc.edu/methods/mri-tool/mri-analysis/).

### Amyloid PET

Amyloid PET imaging was undertaken using AV-45. Amyloid images were acquired in 4 × 5 min frames starting 50–70 min after intravenous bolus injection. The detailed acquisition procedures were described in ADNI PET Technical Procedures Manual (https://adni.loni.usc.edu/methods/pet-analysis-method/pet-analysis/).

Global Aβ burden was represented using the mean florbetapir uptake in gray matter within lateral and medial frontal, anterior, and posterior cingulate, lateral parietal, and lateral temporal regions relative to uptake in the whole cerebellum (white and gray matter) for each subject. Aβ status was assessed based on global Aβ burden, using pre-established protocols and cut-points (global AV45 SUVR > 1.11).

### Imaging analysis

#### WMH segmentation

T2 FLAIR images were used for WMH lesion segmentation. An automatic segmentation tool (Lesion Segmentation Tool) using a lesion prediction algorithm (LPA) based on Statistical Parametric Mapping software (SPM12, http://www.fil.ion.ucl.ac.uk/spm) was used [34]. The automatically created WMH label images were then manually corrected for scalp and tissues falsely classified as WMH and WMH falsely classified as normal white matter. We extracted segmented WMH volumes with the “extract values of interest” function in LST.

#### The quantification of total intracranial volume (TIV)

TIV was segmented using the Computational Anatomy Toolbox (CAT12, http://dbm.neuro.uni-jena.de/cat/). In brief, T1 images were set as inputs, and then the images were bias-corrected and tissue-classified (GM, white matter (WM) and cerebral spinal fluid (CSF)). We visually checked the segmentation results to ensure the quality.

Finally, the overall TIV was obtained using the CAT12 estimating TIV function.

#### DTI-ALPS evaluation

1. Preprocessing of DTI

The preprocessing of DTI, including Gibbs ringing removal, eddy-current and head motion correction, and bias field correction, was performed using the using MRtrix3 (http://www.mrtrix.org).

2) DTI-ALPS index analysis

**Fig. 1:**
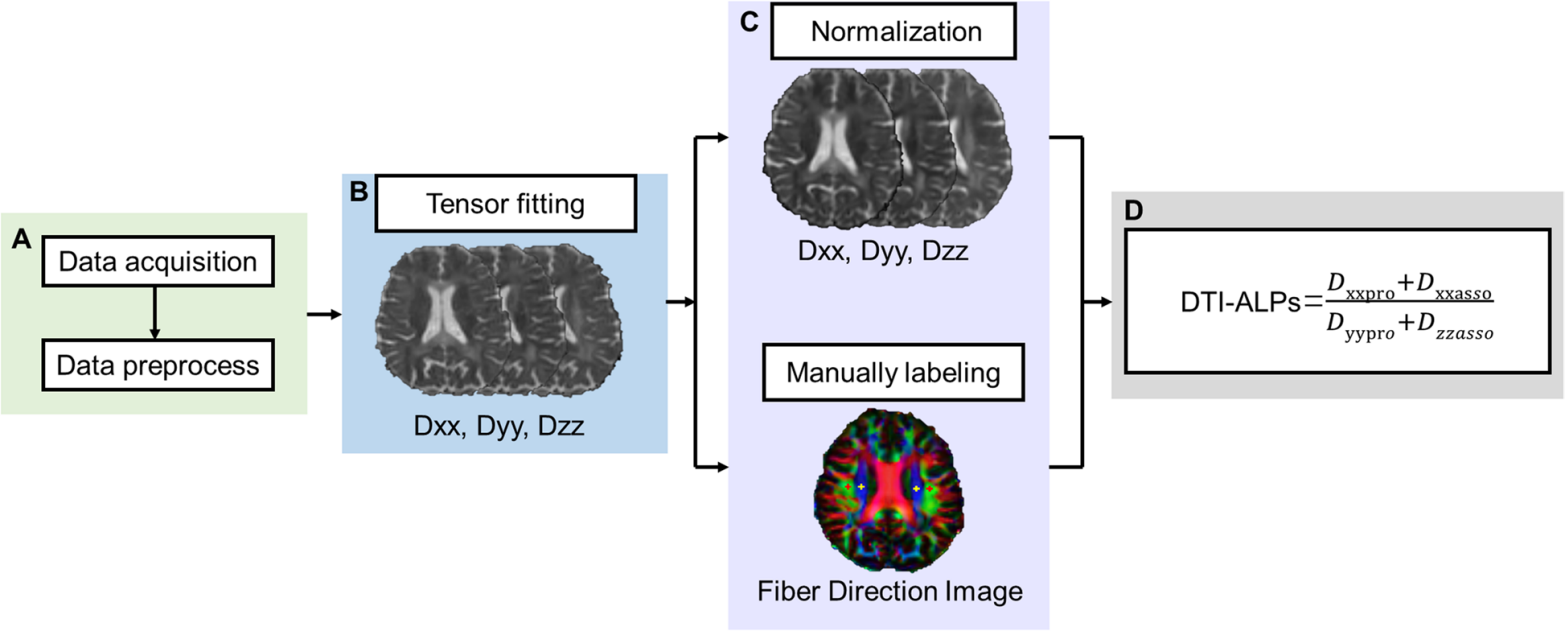
DTI-ALPS processing streamline. **A** Preprocessing of DTI images. **B** DTI Tensor fitting to acquire Dxx, Dyy, and Dzz images. **C** Normalization and manually labeling ROI according to the fiber direction images. **D** Calculation of DTI-ALPS

DTI-ALPS were calculated according to the previous article [18]. Diffusivities maps [Dxx, Dyy, Dzz] acquired from preprocessed DTI were co-registered to the FA map template (JHU-ICBM-FA-2 mm). Four 6-mm-diameter ROIs were designed in the Montreal Neurological Institute (MNI) space. Finally, the manual correction was carried out to confirm the accuracy of registration and the location of ROIs for each participant based on fiber direction image. See details in Fig. 1A–C. As previously noted in a prior study [28], it was suggested that ROIs in the anterior and posterior regions may exhibit distinct regional glymphatic clearance effects. Therefore, we conducted further adjustments by shifting our manually corrected middle ROIs one and two voxels forward and one and two voxels backward along the *y*-axis. Consequently, we derived five groups of ROIs: Anterior 2-voxel ROIs (2), Anterior 1-voxel ROIs (1), Middle ROIs (0), Posterior 1-voxel ROIs (-1), and Posterior 2-voxel ROIs (-2). Left and right DTI-ALPS index was calculated as [(Dxx-proj + Dxx-assoc)/(Dyy-proj + Dzz-assoc)] on each side, respectively. Average DTI-ALPS was calculated as the mean of the left and right DTI-ALPS index. See details in Fig. 1D.

#### PVS rating

The severity of PVS enlargement was rated based on T1 images [35]. PVS enlargement in basal ganglia and white matter region were rated separately. The detailed rating are as follows: in basal ganglia, PVS enlargement severity is rated 1 when there were < 5 PVS, rated 2 when there were 5 ~ 10, > 10 PVS but still countable then rated 3, and rated 4 when the number is uncountable; in white matter region, PVS enlargement severity was rated 1 when there were < 10 PVS in total, rated 2 when there were > 10 PVS in total but no more than 10 PVS in a single section, rated 3 when there were 10 ~ 20 PVS in the section containing the greatest number of PVS, and rated 4 when there were > 20 PVS in any single section.

#### Choroid plexus volume segmentation

T1 images were segmented automatically based on FreeSurfer version 6.0. The volume of both left choroid plexus and right choroid plexus was acquired for each participant.

#### Groups

The included participants were divided into 4 groups—CN − : cognitive normal with amyloid negative and without severe CSVD (WMH FAZAKAS score of < 2), CN + : cognitive normal with amyloid positive, MCI + : mild cognitive impairment with amyloid positive, AD + : Alzheimer’s dementia with amyloid positive.

#### Statistical analysis

All the statistical analysis was performed using SPSS Statistics, Version 27.0 (IBM), and in R (version 4.2.1). Mean ± SD and frequency (%) were presented for continuous variables and categorical variables, respectively. ANOVA and chi-square tests were performed for demographics among groups. To examine the alterations in DTI-ALPS and choroid plexus volume across the AD continuum, we determined W-scores for Aβ, DTI-ALPS, and choroid plexus volume. This calculation was based on reference data from CN − . To enhance visualization, DTI-ALPS was multiplied by − 1 when computing the *W*-score. Subsequently, we conducted nonlinear curve fitting across the AD continuum. The specific details of the *W*-score calculation can be found in Supplementary Material 3. The primary statistical analyses encompassed participants with amyloid positive along the AD continuum and included the following components:

Partial correlation analysis was carried out to analyze the independent relationship of global Aβ burden and WMH with DTI-ALPS and choroid plexus volume. The “clmm” function in R was used to investigate the relationship of global Aβ burden and WMH with basal ganglia PVS and white matter PVS rating score. In the first model, we firstly set age, sex, and TIV as covariates. In model 2, we additionally include APOE ε4 carrier status (APOE ε4 carrier status was coded dichotomously, with ε4 noncarriers coded as 0 and carriers coded as 1) as covariates. Because APOE ε4-alleles had been demonstrated to be associated with the severity of PVS in previous studies [36, 37]. Partial correlation analysis was conducted to investigate the relationship between DTI-ALPS, choroid plexus volume, basal ganglia PVS, and white matter PVS and cognitive performance in each domain. We employed the Spearman method for basal ganglia PVS and white matter PVS analysis. In contrast, we utilized the Pearson method for DTI-ALPS and choroid plexus volume-related analysis. Age, sex, education, and APOE ε4 carrier status were set as covariates. The false discovery rate (FDR) correction method was used for multiple comparison correction.

Mediation analyses were carried out to explore the mediating role of DTI-ALPS and choroid plexus volume on the relationship between WMH volume or global Aβ burden and cognitive performances. Detailly, the mediation analysis was performed using R “rodmed” package [38]. WMH volume and global Aβ burden were included as independent variables, and age, sex, and APOE ε4 carrier status were set as covariates. A 95% bootstrap confidence interval based on 5000 bootstrap replicates was used to estimate significance. FDR correction method was used for multiple comparison correction.

## Results

### Participants

**Fig. 2:**
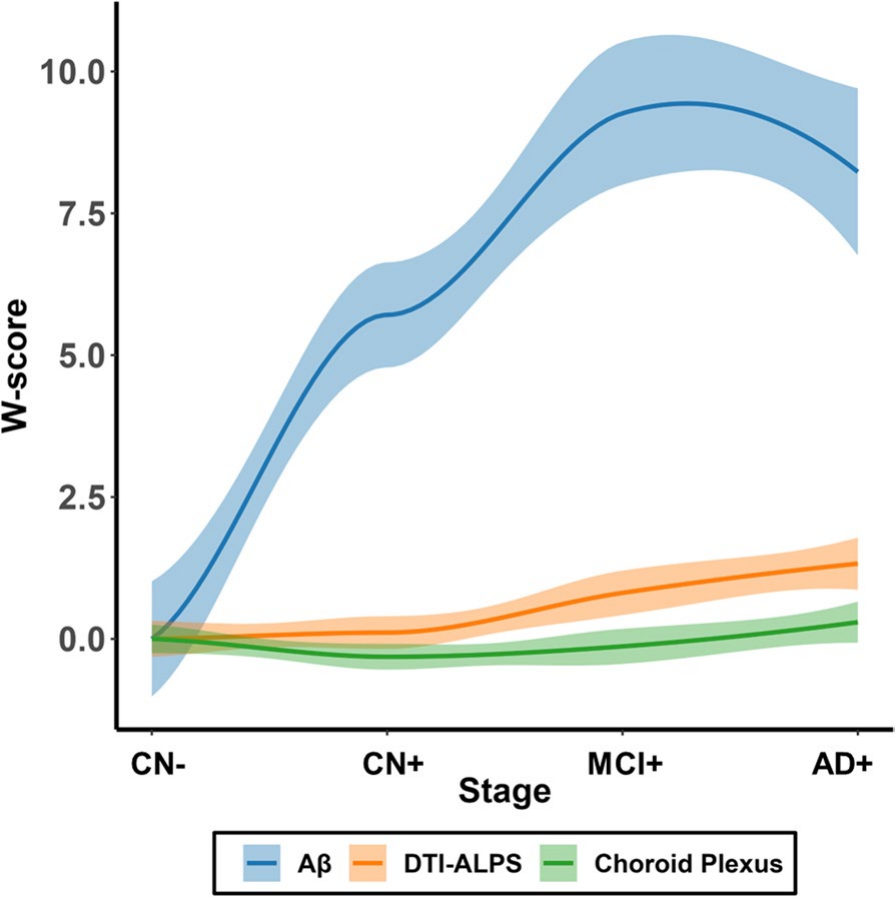
Nonlinear curve fitting of global amyloid aggregation, DTI-ALPS and choroid plexus volume along AD continuum

In this study, we finally included 133 participants. Table 1 shows the demographics of 40 CN A − , 48 CN A + , 26 MCI A + , and 19 AD A + participants. There were no significant differences in age, sex, years of education, TIV, and APOE ε4 carrier status among groups. The global Aβ burden, DTI-ALPS, choroid plexus volume, and WMH burden showed significant differences among groups. However, there were no significant difference for basal ganglia PVS rating score and white matter region rating score. Curve-fitting results of Aβ aggregation DTI-ALPS and choroid plexus volume along the AD continuum are shown in Fig. 2. The abnormality of DTI-ALPS and choroid plexus volume consistently increased from CN A + stage to AD A + stage dementia, while Aβ aggregated from the CN A − stage to MCI A + stage.

**Table 1:** Demographic data of participants along AD continuum

	**CN A − (*N* = 40)**	**CN A + (*N* = 48)**	**MCI A + (*N* = 26)**	**AD A + (*N* = 19)**	***p***
**Demographics**					
Age (years)	75.2 ± 8.4	74.9 ± 7.5	75.4 ± 9.2	75.7 ± 9.6	0.123
Male, *N* (%)	17 (42.5%)	17 (35.4%)	7 (26.9%)	9 (47.4%)	0.367
Education (years)	16.1 ± 2.4	16.6 ± 2.4	15.8 ± 2.3	15.3 ± 2.4	0.144
APOE ε4, *N* (%)	9 (23.1%)^a^	22 (45.8%)^a^	15 (57.7%)	11 (57.9%)	0.763
**Vascular risk factors**					
Hypertension, *N* (%)	14 (35.0%)	22 (45.8%)	13 (50.0%)	8 (44.4%)	0.630
Diabetes, *N* (%)	3 (7.5%)	6 (12.5%)	0	0	0.128
Hyperlipidemia, *N* (%)	16 (40%)	26 (54.2%)	12 (46.2%)	8 (44.4%)	0.608
Smoking, *N* (%)	6 (15.0%)	9 (18.8%)	6 (23.1%)	1 (5.6%)	0.458
Heart disease, *N* (%)	6 (15.0%)	4 (8.3%)	4 (15.4%)	3 (16.7%)	0.701
**Imaging characteristics**					
TIV (mm^3^)	1483.4 ± 147.4	1461.7 ± 150.0	1457.2 ± 147.0	1467.4 ± 207.8	0.901
WMH burden	− 0.006 ± 0.505	0.391 ± 0.723	0.418 ± 0.646	0.868 ± 0.532	< 0.001
Presence of lacune, *N* (%)	2 (5%)	5 (10.4%)	2 (7.7%)	1 (5.3%)	0.781
Presence of microbleed (%)	4 (10%)	3 (6.3%)	3 (11.5%)	6 (31.6%)	0.036
Global Aβ burden (SUVR)	1.024 ± 0.047	1.298 ± 0.178	1.458 ± 0.201	1.417 ± 0.161	< 0.001
PVS rating score					
Basal ganglia PVS	2.00 (2.00–3.75)	2.50 (2.00–3.00)	3.00 (1.00–3.25)	3.00 (1.00–4.00)	0.041
White matter PVS	2.00 (2.00–2.75)	3.00 (2.00–3.00)	2.00 (1.75–3.00)	3.00 (2.00–3.00)	0.987
Choroid plexus volume	1.085 ± 0.339	1.005 ± 0.244	1.061 ± 0.232	1.229 ± 0.225	0.029
DTI-ALPS	1.355 ± 0.155	1.341 ± 0.106	1.255 ± 0.161	1.154 ± 0.184	< 0.001

The log-transformed WMH volume is used to represent the burden of white matter hyperintensity (WMH)

The choroid volume (mm^3^)/TIV (ml)*10^3^ is used to represent choroid plexus volume

*CN *Cognitive normal, *MCI *Mild cognitive impairment, *AD *Alzheimer’s disease, *TIV *Total intracranial volume, *WMH *White matter hyperintensity, *PVS *Perivascular space

^a^One CN − and 4 CN + participants without the information of APOE status

We did not detect a significant difference among DTI-ALPS in different locations (Supplementary Material 4), and the correlation between DTI-ALPS in each location was consistently high (Supplementary Material 5). As a result, we opted to utilize the DTI-ALPS from the middle ROIs for all our analyses.

### Associations of glymphatic markers with Aβ accumulation and WMH burden on amyloid positive participants

**Fig. 3:**
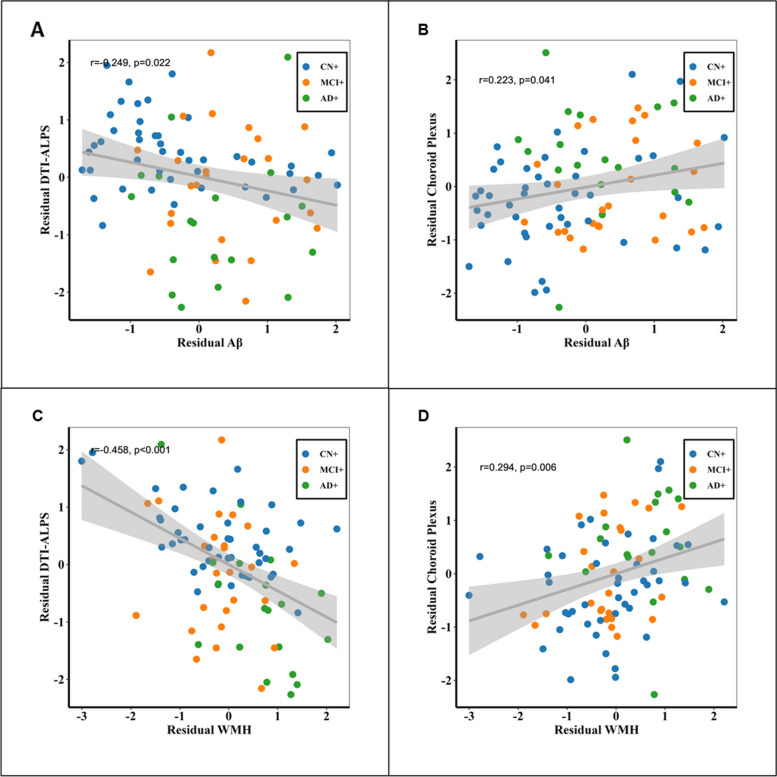
The association of DTI-ALPS and choroid plexus volume with WMH burden and Aβ in amyloid positive participants. **A** The association between Aβ and DTI-ALPS. Residual Aβ and residual DTI-ALPS represented amyloid burden and DTI-ALPS corrected by age, sex, TIV, and APOE status. **B** The association between Aβ and choroid plexus volume. Residual Aβ and residual Choroid Plexus represented amyloid burden and DTI-ALPS corrected by age, sex, TIV, and APOE status. **C** The association between WMH burden and DTI-ALPS. Residual WMH and residual DTI-ALPS represented WMH burden and DTI-ALPS corrected by age, sex, TIV, and APOE status. **D** The association between WMH burden and choroid plexus. Residual WMH and residual choroid plexus represented WMH burden and DTI-ALPS corrected by age, sex, TIV, and APOE status

We observed a negative association between global Aβ burden and DTI-ALPS (*r* =  − 0.240, *p* = 0.023) and a positive correlation with choroid plexus volume (*r* = 0.217, *p* = 0.041) after adjusting for the effects of age, sex, and TIV. These associations remained significant when including APOE ε4 carrier status as an additional covariate (*r* =  − 0.249, *p* = 0.022) for DTI-ALPS and (*r* = 0.223, *p* = 0.041) for choroid plexus volume, as illustrated in Fig. 3A and B, respectively.

Regarding WMH burden, we found it to be negatively associated with DTI-ALPS (*r* =  − 0.449, *p* < 0.001) and positively correlated with choroid plexus volume (*r* = 0.280, *p* < 0.001) after correcting for age, sex, and TIV. These associations remained significant even when additionally correcting for the effects of APOE ε4 carrier status (*r* =  − 0.458, *p* < 0.001) for DTI-ALPS (Fig. 3C) and (*r* = 0.294, *p* = 0.006) for choroid plexus volume (Fig. 3D).

However, for both basal ganglia PVS and white matter region PVS, we did not observe any significant correlation with global Aβ burden or WMH burden, regardless of whether we included APOE status as a covariate, as shown in Table 2.

**Table 2:** The association of basal ganglia PVS and white matter PVS with WMH burden and Aβ in amyloid positive participants

	Basal ganglia PVS		White matter PVS	
	*β*	*p*	*β*	*p*
Model 1				
Aβ	− 1.238	0.347	− 0.867	0.494
WMH burden	0.542	0.173	0.054	0.889
Model 2				
Aβ	− 1.212	0.379	− 0.754	0.575
WMH burden	0.489	0.225	− 0.054	0.890

Model 1: age, sex, and TIV were included as covariates

Model 2: age, sex, TIV, and APOE status were included as covariates

*TIV *Total intracranial volume, *WMH *White matter hyperintensity, *PVS *Perivascular space

Considering the potential impact of vascular risk factors on glymphatic markers, we replicated the aforementioned analysis specifically in participants with at least one vascular risk factor. Additionally, we conducted correlations between glymphatic markers and amyloid/WMH, with an additional correction for the vascular risk factor score. Both analyses showed that the association between amyloid/WMH and glymphatic markers (DTI-ALPS and choroid plexus volume) remained significant. Further details can be found in Supplementary Material 6 and 7. Furthermore, we explored the comparison of participants with and without each vascular risk factor. Notably, participants with heart disease exhibited a significant decrease in DTI-ALPS (FDR-*p* = 0.012). However, no significant results were found for correlations with the other vascular risk factors. Additional details are provided in Supplementary Material 8.

### Associations between glymphatic markers and cognitive performance on amyloid positive participants

In our partial correlation analysis, we observed significant associations between DTI-ALPS and cognitive domains. DTI-ALPS showed a positive association with memory (*r* = 0.470, FDR-*p* < 0.001), executive function (*r* = 0.358, FDR-*p* = 0.001), visual-spatial (*r* = 0.223, FDR-*p* < 0.040), and language (*r* = 0.419, FDR-*p* < 0.001).

Similarly, we found significant correlations between choroid plexus volume and cognitive domains, with choroid plexus volume showing a negative association with memory (*r* =  − 0.315, FDR-*p* = 0.007), executive function (*r* =  − 0.321, FDR-*p* = 0.007), visual-spatial (*r* =  − 0.233, FDR-*p* = 0.031), and language (*r* =  − 0.261, FDR-*p* = 0.021).

However, there were no significant correlations between basal ganglia PVS burden and white matter PVS burden with cognitive performance. Further details can be found in Table 3.

**Table 3:** The association between glymphatic markers and cognitive domains

	DTI-ALPS		Choroid plexus		Basal ganglia PVS		White matter PVS	
	*r*	FDR-p	*r*	FDR-p	Rho	FDR-p	Rho	FDR-p
Memory	0.470	< 0.001	− 0.315	0.007	0.060	0.704	0.054	0.788
Executive function	0.358	0.001	− 0.321	0.007	− 0.042	0.704	0.114	0.601
Visual-spatial	0.223	0.040	− 0.233	0.031	− 0.076	0.704	− 0.030	0.261
Language	0.419	< 0.001	− 0.261	0.021	− 0.082	0.704	0.201	0.788

*PVS *Perivascular space, *FDR *False discovery rate

### Glymphatic markers as a significant mediator between Aβ accumulation/and WMH burden cognitive performance on amyloid positive participants

**Fig. 4:**
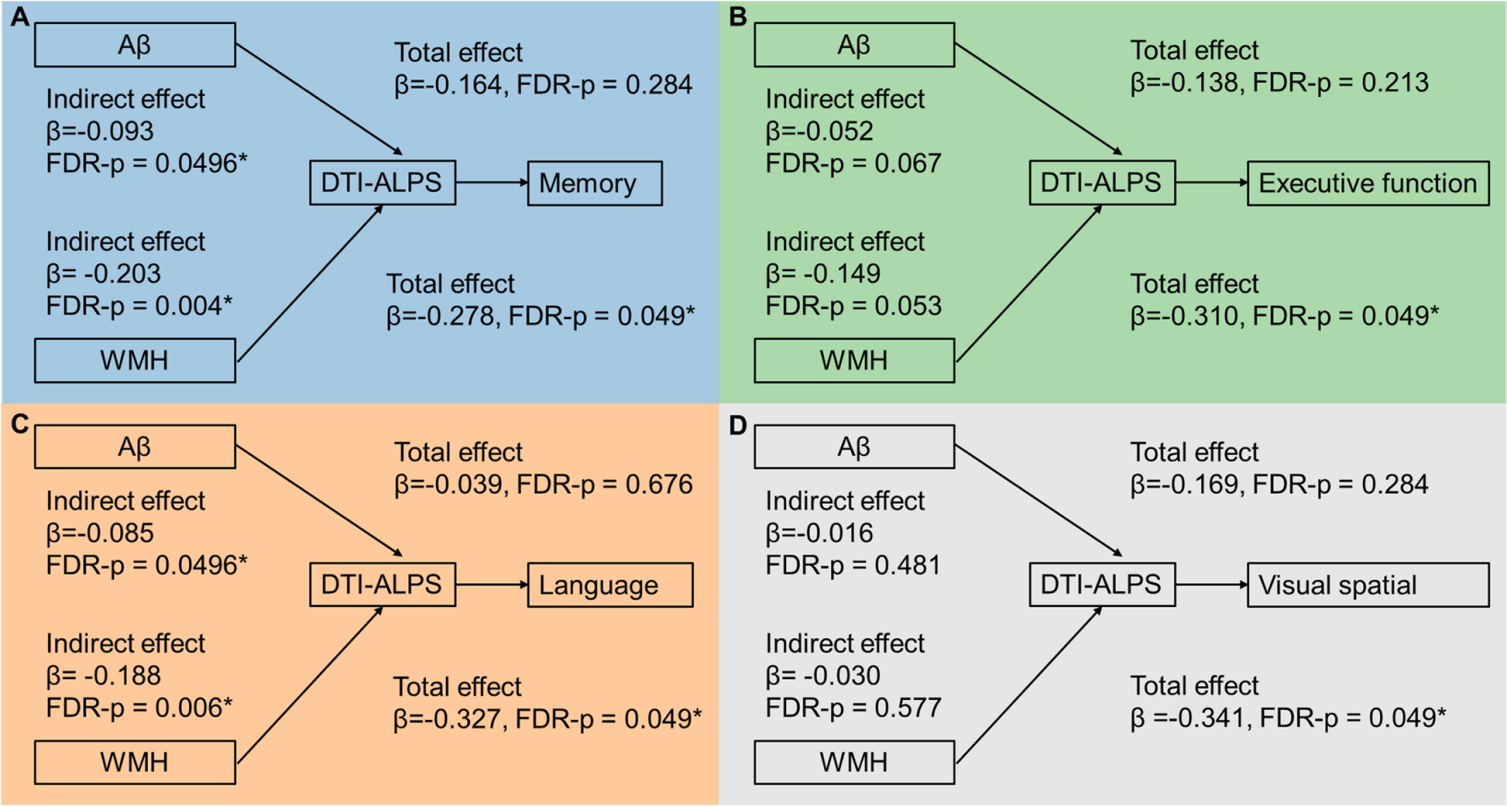
The mediating effect of DTI-ALPS in the relationship between WMH burden/Aβ accumulation and cognitive performance

In the mediation analysis, DTI-ALPS also showed significant mediating effects on the relationship between whole brain Aβ accumulation and memory (indirect effects *β* =  − 0.093, FDR-*p* = 0.0496) and language performance (indirect effects *β* =  − 0.085, FDR-*p* = 0.0496). However, we did not find the mediating role of DTI-ALPS on the relationship between Aβ and executive function (*β* =  − 0.052, FDR-*p* = 0.067) and visual-spatial performance (indirect effects *β* =  − 0.169, FDR-*p* = 0.284), after FDR multiple comparison correction. DTI-ALPS showed significant mediation effects between WMH burden and memory (indirect effects *β* =  − 0.203, FDR-*p* = 0.004) and language performance (indirect effects *β* =  − 0.188, FDR-*p* = 0.006). We did not find the mediating role of DTI-ALPS on the relationship between WMH burden and executive function (indirect effects *β* =  − 0.149, FDR-*p* = 0.053) and visual-spatial performance (indirect effects *β* =  − 0.041, FDR-*p* = 0.354) after FDR correction. However, the total effects of WMH burden on both executive function (total effects *β* =  − 0.149, *p* = 0.049) and visual-spatial performance (total effects *β* =  − 0.341, FDR-*p* = 0.049) were significant. See details in Fig. 4.

**Fig. 5:**
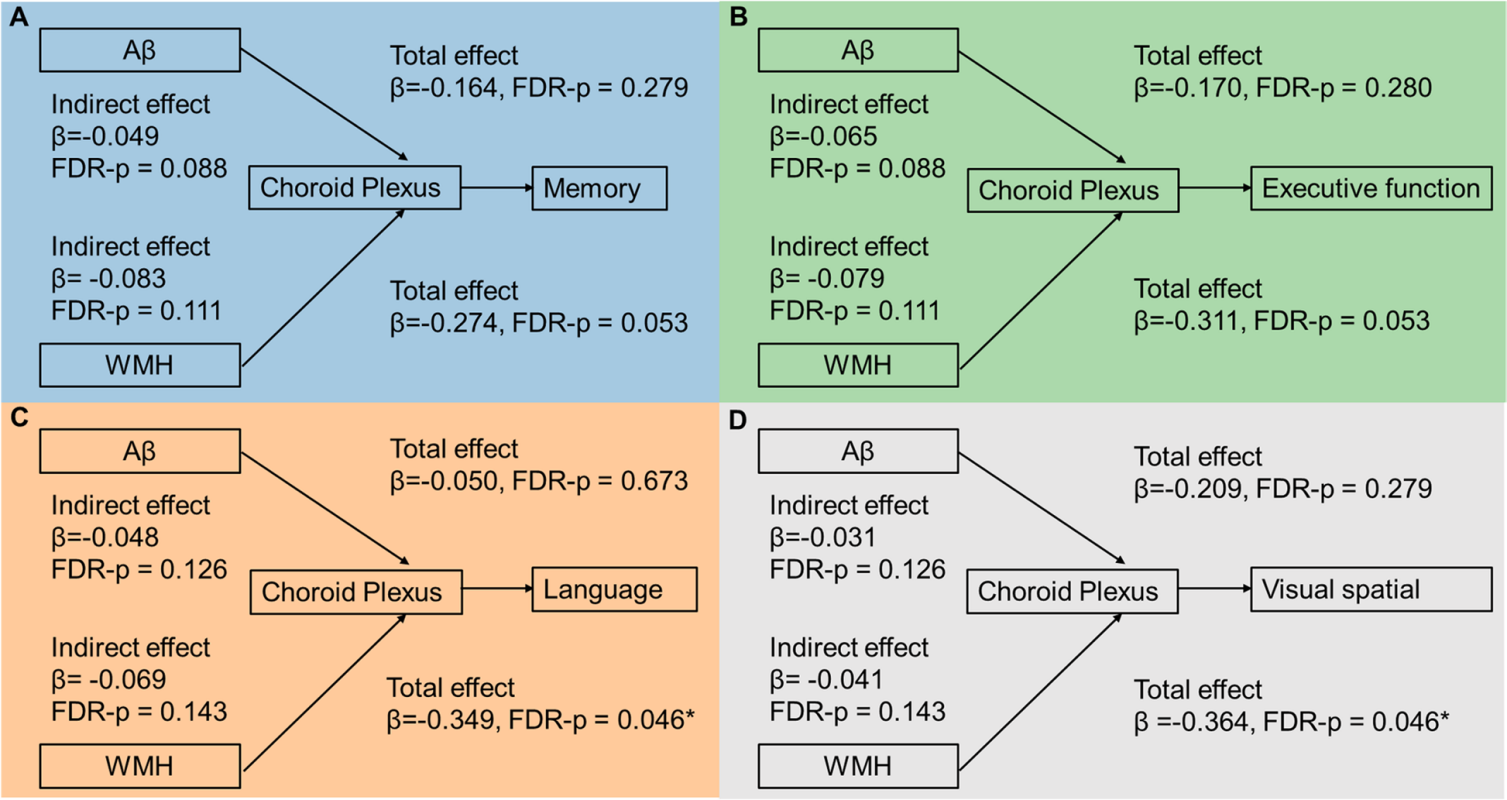
The mediating effect of choroid plexus volume in the relationship between WMH burden/Aβ accumulation and cognitive performance

Regarding choroid plexus volume, our analysis did not reveal any significant indirect effects for both Aβ accumulation and WMH burden on each cognition sub-domain after applying FDR multiple comparison correction, as depicted in Fig. 5.

## Discussion

This study found that (1) both CSVD and Aβ burden were independently associated with DTI-ALPS and choroid plexus volume; (2) cognitive performance in most domains was associated with DTI-ALPS and choroid plexus volume; and (3) more importantly, DTI-ALPS mediated the relationship between CSVD/Aβ and cognitive performance.

A series of studies have demonstrated the association between Aβ and glymphatic function [3, 5, 6, 39]. Our study using in vivo imaging methods found that decreased DTI-ALPS and increased choroid plexus volume were related to increased Aβ aggregation. However, we did not find the correlation between PVS enlargement severity and Aβ aggregation, which was consistent with previous findings [40, 41]. DTI-ALPS assesses diffusivity along deep medullary veins and is suggested as a marker for evaluating glymphatic clearance function [17]. Choroid plexus volume plays a crucial role in the production of cerebrospinal fluid (CSF) and may act as a driving force for the glymphatic system [13]. Enlarged PVS observed on MRI is mostly situated around arterioles, which might cause hydrodynamic changes in CSF inflow and exchange with parenchymal fluid [42]. The differential association of these markers with Aβ aggregation may suggest distinct contribution of the sub-processes in the waste clearance pathway. Nonetheless, it could also be due to different sensitivities of these MRI methods. Further validations are still needed.

By plotting the changes of DTI-ALPS and choroid plexus volume against amyloid accumulation along AD continuum, we found that Aβ accumulated from CN − to MCI + and flattened afterwards, whereas both DTI-ALPS and choroid plexus volume exhibit consistent changes from CN + to AD + . Notably, the curve representing Aβ accumulation exhibited an earlier onset compared to the changes in DTI-ALPS and choroid plexus volume. These results seem to be inconsistent from previous assumptions that amyloid deposition should be a consequence of glymphatic dysfunction. Indeed, the evidence concerning the association between glymphatic dysfunction and Aβ accumulation has primarily been derived from comparative studies. For example, APP mice showed decreased glymphatic function than wild mice [5, 43], and late-onset AD participants demonstrated glymphatic impairment compared with normal controls [39]. However, it is essential to acknowledge that comparing AD and non-AD is not sufficient to conclude that glymphatic dysfunction results in Aβ aggregation or vice versa. The complexities of this relationship and the direction of causality indeed call for further investigation to achieve a more comprehensive understanding. Some studies using animal models with deficiency of AQP-4 [3, 6], which was suggested as the most important channel for glymphatic function, suggested that glymphatic dysfunction was followed by Aβ aggregation. However, it is important to consider that brain parenchymal Aβ plaques could also drive reduced perivascular localization of AQP-4 [44, 45], and this would cause impaired glymphatic function. Besides, Aβ distributed in the walls of capillaries, and arteries could impair the motion for fluid movement along perivascular space [46, 47], therefore decreasing the fluid diffusivity. Moreover, Aβ-related inflammatory reactions might increase resistance to fluid movement in perivascular space [48]. Two studies also indicated the late-stage involvement of in vivo glymphatic markers [8, 40]. One of these studies emphasized the relationship between AD pathologies and DTI-ALPS, suggesting that DTI-ALPS may be a later marker than AD pathologies. Another study, which focused on the severity of PVS burden, also did not find evidence to support the role of in vivo glymphatic markers in the early stages of AD pathogenesis [40]. These collective findings emphasize the complex and multifaceted nature of AD progression, requiring a deeper exploration of the temporal relationships among various factors involved in the disease.

Our finding of the association between CSVD and glymphatic markers in the AD continuum was in line with previous studies focusing on CSVD patients [13, 18, 21]. Brain-wide network of perivascular pathways made up glymphatic system [49], so the damage of small cerebral vessels was closely correlated with glymphatic function. Fluid movement along perivascular space depends on the pulsation of the cerebral artery. Studies on cerebral arterial pulsation found that arterial stiffness was associated with glymphatic dysfunction [23, 50, 51]. Also, venous collagenosis was suggested to be associated with decreased glymphatic clearance function [52, 53]. Apart from the motion provided by cerebral small vessels, the blood–brain barrier (BBB) broken with increased perivascular inflammation factors and wastes could increase resistance to fluid motion. AQP-4 was also reduced in CSVD rat models [10, 54]. Taken together, CSVD-related decreased driving force, increased resistance, and loss of water channel may all lead to damaged glymphatic function along the AD continuum.

We further found an association of DTI-ALPS and choroid plexus volume with cognitive performance in each domain. This result responded to the opinion that “glymphatic failure is a final common pathway to dementia” [2]. Unlike previous studies, we considered the effect of CSVD on cognition that could be mediated by glymphatic function, in addition to the effect of Aβ along the AD continuum. We observed that the relationship of CSVD and Aβ with memory and language was mediated by DTI-ALPS, and the indirect effect coefficient was the largest in the memory domain. This finding corroborated the idea that AD dementia is multifactorial rather than just AD pathologies. Another interesting observation was that though we did not find glymphatic markers as the mediator between CSVD and visual-spatial performance and executive function, the total effects were significant, which meant that CSVD had another way to impact AD progression. This notion further supported the pivotal role of CSVD along the AD continuum [55, 56].

Our study helps to further understand the role of the in vivo glymphatic index in AD. Firstly, unlike previous studies that only focused on AD pathologies in AD dementia or CSVD on vascular dementia, our study investigated the independent or interactive effects of AD pathologies and CSVD on AD continuum participants, consisting of participants from CN to dementia. The results of the independent effects of both pathologies suggested that controlling Aβ or CSVD could effectively improve glymphatic function. More importantly, our finding from the view of glymphatic function added the mechanism-based evidence for mixed AD dementia (AD pathologies combined with CSVD), in addition to prior studies focusing on their addictive role on whole brain functional or structural connectivity.

Our study had several limitations. Firstly, our evaluation of CSVD was solely based on WMH burden, without considering other important CSVD markers such as lacunes and microbleeds. It is important to acknowledge that WMH can have non-vascular origins, which might potentially introduce some bias into our results. However, it is worth noting that most pathogenic studies have primarily associated WMH with CSVD. Furthermore, the low prevalence of lacunes (12%) and microbleeds (7.5%) among our study participants had a substantial impact on the statistical power of our analysis. Secondly, our study involved participants from 17 different centers, which could introduce variability in DTI-ALPS. Due to the small sample size, it is difficult to compare participants from different centers. Nonetheless, as the acquisition protocol of ADNI has been harmonized by a specialized imaging core, the difference should have been minimized between these SIEMENS scanners. Lastly, our study was cross-sectional in nature. Although our hypothesis was supported by prior evidence, curve fitting along the AD continuum, and mediation analysis, a longitudinal design would be more suitable for a more comprehensive exploration of this question. These limitations should be considered when interpreting our findings, and they also point to opportunities for future research to address these issues and provide a more robust understanding of the relationship between CSVD, AD biomarkers, and glymphatic markers.

In conclusion, our study provided evidence that AD pathology (Aβ) and CSVD were associated with glymphatic dysfunction, which is further related to cognitive impairment.

### Supplementary Information

**Additional file 1:** **Supplementary Material 1.** Demographics of participants with and without chin-up. **Supplementary Material 2.** Methods. **Supplementary Material 3.** **Supplementary Material 4.** Comparison of DTI-ALPS among different locations. **Supplementary Material 5.** The correlations between different locations DTI-ALPS. **Supplementary Material 6.** The association of DTI-ALPS and Choroid Plexus volume with WMH burden and Aβ in amyloid positive participants with at least one vascular risk factor. **Supplementary Material 7.** The association of DTI-ALPS and Choroid Plexus volume with WMH burden and Aβ in amyloid positive participants corrected for vascular risk factor score. **Supplementary Material 8.** Glymphatic markers comparison between participants with and without each vascular risk factor in amyloid positive participants.

## Data Availability

The datasets generated and/or analyzed during the current study are available in the ADNI repository, http://www.adni-info.org.
