# Supplementary material for: The relationship between amyloid pathology, cerebral small vessel disease, glymphatic dysfunction, and cognition: a study based on Alzheimer’s disease continuum participants
Source: Alzheimers Res Ther. 2024 Feb 20;16:43. doi: 10.1186/s13195-024-01407-w (PMC10877805; doi:10.1186/s13195-024-01407-w)

**Supplementary Material 1**.

**Demographics of participants with and without chin-up**

| **Demographics** | **Participants with chin-up**  (n=20) | **Participants without chin-up**  (n=133) | ***p*** |
| --- | --- | --- | --- |
| Age (years) | 79.8±8.4 | 74.1±8.1 | 0.004 |
| Male, N (%) | 15(75%) | 45(33.8%) | 0.001 |
| Education (years) | 17.5±2.7 | 16.2±2.3 | 0.032 |
| APOE ԑ4, N (%) | 4(20%) | 57(42.9%) | 0.079 |
| **Imaging characteristics** |  |  |  |
| TIV (mm^3^) | 1574.0±116.8 | 1468.1±156.6 | 0.004 |
| WMH burden | 0.602±0.639 | 0.345±0.677 | 0.113 |
| Global Aβ burden (SUVR) | 1.242±0.235 | 1.266±0.230 | 0.666 |
| DTI-ALPS | 1.008±0.165 | 1.302±0.160 | <0.001 |
| **Stage of participants** |  |  | 1.000 |
| CN A-, N (%) | 6(30%) | 40(30.1%) |  |
| CN A+, N (%) | 7(35%) | 48(36.1%) |  |
| MCI A+, N (%) | 4(20%) | 26(19.5%) |  |
| AD A+, N (%) | 3(15%) | 19(14.3%) |  |

CN, cognitive normal; MCI, mild cognitive impairment; AD, Alzheimer's disease; TIV, total intracranial volume; WMH, white matter hyperintensity

**Supplementary Material 2**.

**Methods**

ADNI-Mem was obtained by recording some items. These items included RVLT (Trial 1, Trial 2, Trial 3, Trial 4, Trial 5, Interference, Immediate recall, 30 minutes delay and Recognition), ADAS-Cog (Trial 1, Trial2, Trial 3, Recall, Recognition present and Recognition absent), Logical Memory (Immediate and Delay) and MMSE (Ball recall, Flag recall and Tree recall).

The model for ADNI-EF included Category Fluency-animals, Category Fluency-vegetables, Trails A and B, Digit span backwards, WAIS-R Digit Symbol Substitution, and 5 Clock Drawing items (circle, symbol, numbers, hands, time).

The tests that make up ADNI-Lan included Neuropsychological Battery(Category Fluency-Animals, Category Fluency-Vegetables and Boston Naming), ADAS-Cognitive Behavior (Following Commands, Object Naming and Ideational Practice), MMSE (Naming an Object–Watch, Naming an Object–Pencil, Repeating a Sentence, Reading a Sentence, Writing a Sentence, Following a Series of Instructions) and MoCA (Letter F Fluency, Animal Naming-Lion, Camel, Rhino and Sentence Repetition).

ADNI-VS was consisted by Neuropsychological Battery (Clock copy–Circle, Clock copy–Symmetry, Clock copy–Numbers, Clock copy–Hands, Clock copy–Time), ADAS-Cognitive Behavior (Constructional praxis) and MMSE (Copy design).

**Supplementary Material 3**.

Firstly, we ran linear regression model on CN participants with amyloid negative (A-) and without severe CSVD (defined as participants with FAZAKAS score < 2, V-), for global Aβ burden, Choroid Plexus volume and DTI-ALPS, we regressed age and sex respectively.

Next, we calculated the predicted global Aβ burden, Choroid Plexus volume, and DTI-ALPS for all participants using these regression β-weights derived from CN A-V- participants.

Finally, participants W-scores were then calculated based on the discrepancy between observed and predicted scores and divided by the test-specific control group’s standard error of estimate.

**Supplementary Material 4**. Comparison of DTI-ALPS among different locations

**
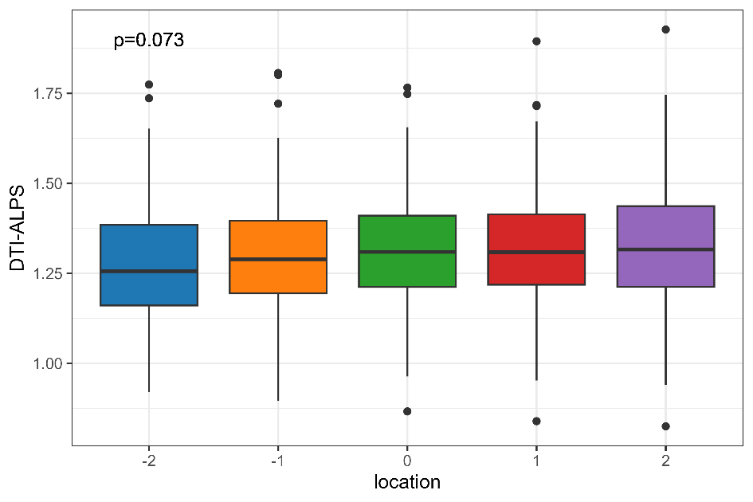
**

2, Anterior 2-voxel ROIs; 1 Anterior 1-voxel ROIs; 0, Middle ROIs; -1, Posterior 1-voxel ROIs; -2, Posterior 2-voxel ROIs.

**Supplementary Material 5**. The correlations between different locations DTI-ALPS


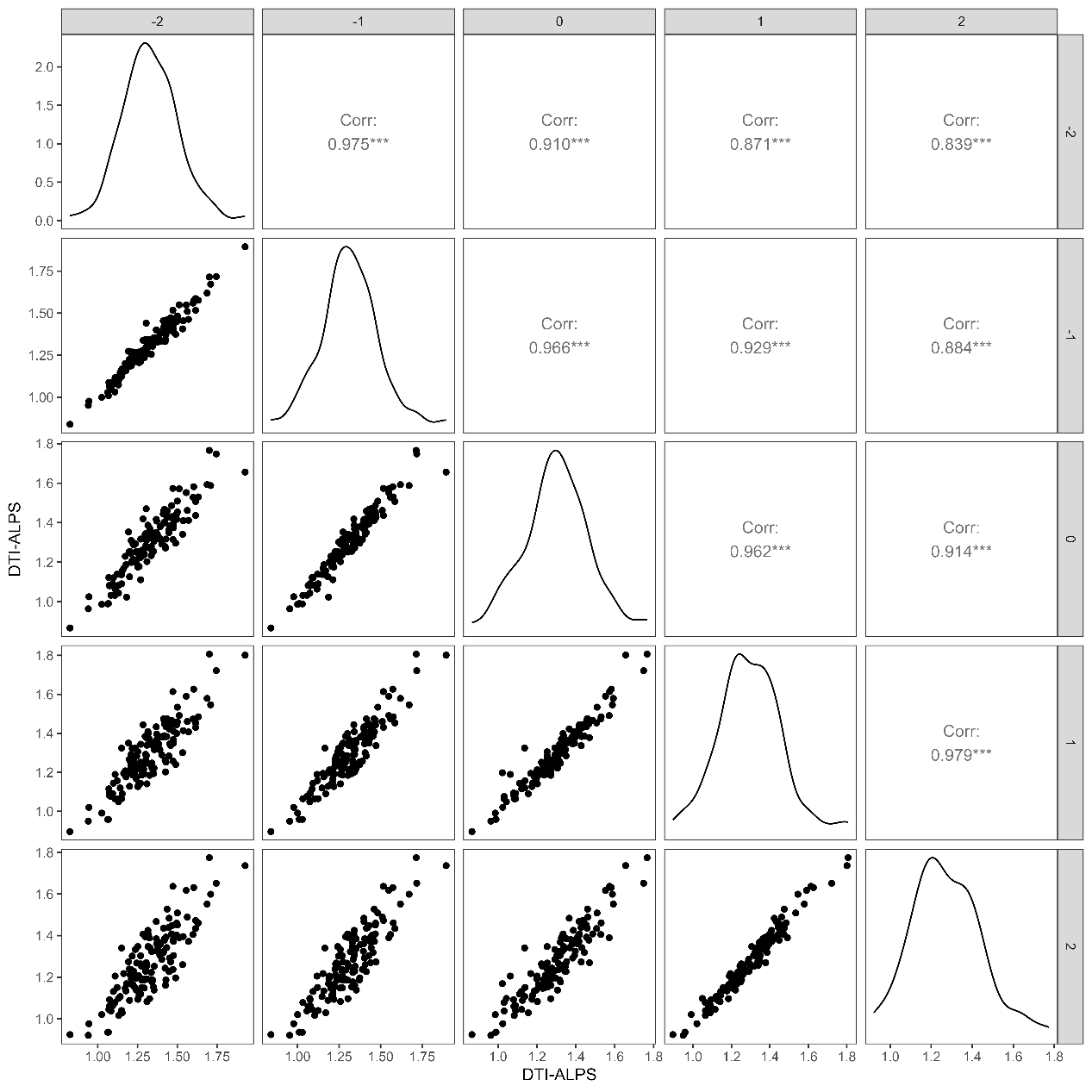


**Supplementary Material 6**. The association of DTI-ALPS and Choroid Plexus volume with WMH burden and Aβ in amyloid positive participants with at least one vascular risk factor

|  | DTI-ALPS |  | Choroid plexus |  |
| --- | --- | --- | --- | --- |
|  | β | p-value | β | p-value |
| WMH burden | -0.461 | <0.001 | 0.400 | 0.002 |
| Aβ | -0.274 | 0.037 | 0.364 | 0.004 |

**Supplementary Material 7**. The association of DTI-ALPS and Choroid Plexus volume with WMH burden and Aβ in amyloid positive participants corrected for vascular risk factor score

|  | DTI-ALPS |  | Choroid plexus |  |
| --- | --- | --- | --- | --- |
|  | β | p-value | β | p-value |
| WMH burden | -0.426 | <0.001 | 0.303 | 0.005 |
| Aβ | -0.222 | 0.045 | 0.234 | 0.034 |

The Vascular risk factor score is calculated by summing the presence of hypertension, diabetes, hyperlipidemia, smoking, and heart diseases.

**Supplementary Material 8**. Glymphatic markers comparison between participants with and without each vascular risk factor in amyloid positive participants


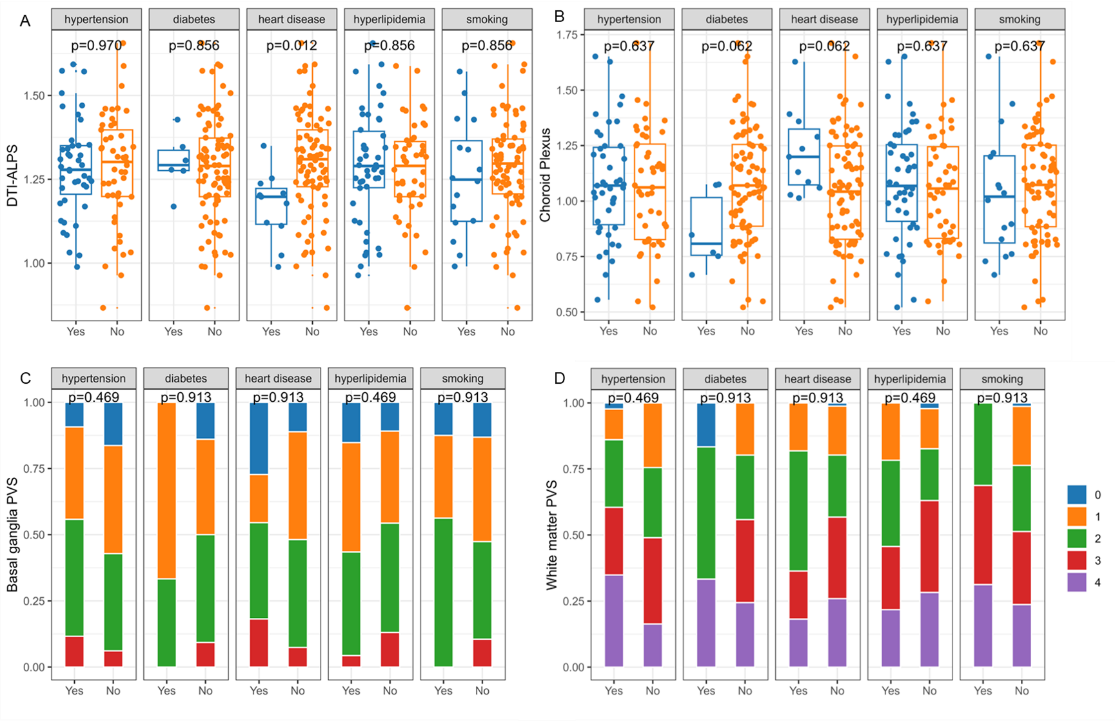

Supplement: Supplementary file 1 — Additional file 1: Supplementary Material 1. Demographics of participants with and without chin-up. Supplementary Material 2. Methods. Supplementary Material 3. Supplementary Material 4. Comparison of DTI-ALPS among different locations. Supplementary Material 5. The correlations between different locations DTI-ALPS. Supplementary Material 6. The association of DTI-ALPS and Choroid Plexus volume with WMH burden and Aβ in amyloid positive participants with at least one vascular risk factor. Supplementary Material 7. The association of DTI-ALPS and Choroid Plexus volume with WMH burden and Aβ in amyloid positive participants corrected for vascular risk factor score. Supplementary Material 8. Glymphatic markers comparison between participants with and without each vascular risk factor in amyloid positive participants. [file 13195_2024_1407_MOESM1_ESM.docx]
